# A Bivariate Mixture Model for Natural Antibody Levels to Human Papillomavirus Types 16 and 18: Baseline Estimates for Monitoring the Herd Effects of Immunization

**DOI:** 10.1371/journal.pone.0161109

**Published:** 2016-08-18

**Authors:** Margaretha A. Vink, Johannes Berkhof, Jan van de Kassteele, Michiel van Boven, Johannes A. Bogaards

**Affiliations:** 1 Centre for Infectious Disease Control, National Institute for Public Health and the Environment, Bilthoven, the Netherlands; 2 Department of Epidemiology and Biostatistics, VU University Medical Centre, Amsterdam, the Netherlands; 3 Department of Statistics, Informatics and Modelling, National Institute for Public Health and the Environment, Bilthoven, the Netherlands; Fondazione IRCCS Istituto Nazionale dei Tumori, ITALY

## Abstract

Post-vaccine monitoring programs for human papillomavirus (HPV) have been introduced in many countries, but HPV serology is still an underutilized tool, partly owing to the weak antibody response to HPV infection. Changes in antibody levels among non-vaccinated individuals could be employed to monitor herd effects of immunization against HPV vaccine types 16 and 18, but inference requires an appropriate statistical model. The authors developed a four-component bivariate mixture model for jointly estimating vaccine-type seroprevalence from correlated antibody responses against HPV16 and -18 infections. This model takes account of the correlation between HPV16 and -18 antibody concentrations within subjects, caused e.g. by heterogeneity in exposure level and immune response. The model was fitted to HPV16 and -18 antibody concentrations as measured by a multiplex immunoassay in a large serological survey (3,875 females) carried out in the Netherlands in 2006/2007, before the introduction of mass immunization. Parameters were estimated by Bayesian analysis. We used the deviance information criterion for model selection; performance of the preferred model was assessed through simulation. Our analysis uncovered elevated antibody concentrations in doubly as compared to singly seropositive individuals, and a strong clustering of HPV16 and -18 seropositivity, particularly around the age of sexual debut. The bivariate model resulted in a more reliable classification of singly and doubly seropositive individuals than achieved by a combination of two univariate models, and suggested a higher pre-vaccine HPV16 seroprevalence than previously estimated. The bivariate mixture model provides valuable baseline estimates of vaccine-type seroprevalence and may prove useful in seroepidemiologic assessment of the herd effects of HPV vaccination.

## Introduction

Human papillomavirus (HPV) is among the most prevalent sexually transmitted infections. Persistent infection with high-risk HPV is the necessary cause for the development of cervical cancer, and may also lead to anogenital and oropharyngeal carcinomas [1]. HPV types 16 and 18 are the main focus of current vaccination programs, as these high-risk types are responsible for the majority of cancer cases [2–5].Vaccination against HPV16 and -18 has been introduced in many countries, including the Netherlands, but eligibility is typically restricted to preadolescent girls and uptake is relatively low; approximately 60% of (pre)adolescent girls in the Netherlands have been vaccinated [6]. Vaccination of preadolescents is not expected to have a noticeable impact on cancer incidence within the coming decades. HPV16 and -18 infections can be acquired soon after sexual debut, but the development of cancer after infection may take several decades [7]. To anticipate the population impact of HPV vaccination at an earlier instance, post-vaccine monitoring programs targeting HPV-related surrogate endpoints have been introduced in many countries [8, 9]. Many of these focus on time-trend analyses in the incidence or prevalence of type-specific HPV infections, anogenital warts, and cervical lesions. Serological surveys might also be useful for observing changes in infection dynamics, but serology is still an underutilized tool in HPV monitoring programs.

Serological surveys are relatively inexpensive and only a small amount of serum is necessary to test for antibodies against a variety of pathogens. These surveys can be used for monitoring the antibody levels in vaccinated individuals, and to inform on post-vaccine changes in infection risk in the non-vaccinated population, the so-called herd effect of mass immunization [10, 11]. These aspects are especially relevant for monitoring HPV vaccination, because both the duration of protection against high-risk HPV types and the herd effects of vaccinating against HPV16 and -18 are still unknown. Herd effects may constitute an important aspect of the overall impact of HPV vaccination programs, as demonstrated by the rapid fall in anogenital warts diagnoses in vaccinated as well as non-vaccinated cohorts in countries with as satisfactory uptake of quadrivalent HPV vaccine [12], which includes low-risk HPV types 6 and 11 associated with warts. The extent of indirect protection against high-risk HPV types will likely be smaller than against low-risk types [13], but substantial herd effects are nonetheless predicted for non-vaccinated women as well as men [14, 15].

In principle, monitoring for herd effects against HPV16 and -18 could be integrated with HPV DNA screening for precancerous cervical lesions. The Netherlands will be the first country to adapt their organized screening program on the basis of primary HPV DNA testing, using the cobas^®^ HPV test which detects HPV16 and -18 individually and a pool of 12 other high-risk HPV types [16]. However, organized screening only starts at 30 years of age in the Netherlands, and monitoring in younger cohorts is hampered by the lack of pre-vaccine data that could serve as a benchmark. Moreover, herd effects in men would go unnoticed by reliance on HPV DNA testing in cervical screening. As an alternative, herd effects can be monitored by means of serological surveys, two of which have already been carried out prior to the introduction of HPV vaccination in the Netherlands, with a third one scheduled for 2016/2017. These surveys have been informative in studying infection dynamics of other vaccine-preventable diseases, such as mumps and rubella [17, 18]. An additional advantage regarding HPV is the straightforward attribution of vaccination status, as vaccine-derived antibody levels are approximately 10–100 times higher as compared to naturally derived HPV-specific antibodies [19]. Conversely, changes in antibody levels among non-vaccinated individuals could be employed to infer herd effects of HPV vaccination.

Use of serology for post-vaccine monitoring is complicated, because the antibody response to HPV infection is generally weak. Currently used serological assays are able to accurately quantify vaccine-induced antibody levels, but detection of natural antibodies is difficult due to a poor signal to noise ratio [20]. Consequently, classifying individuals as naturally seropositive for multiple HPV types may suffer from a high misclassification rate. Changes in the population antibody levels may still be detectable, but require an appropriate statistical model to inform about herd effects of HPV vaccination.

Previously, we have shown that a probabilistic assignment based on a mixture model can be used for the estimation of seroprevalence as a function of age [21]. With multi-strain pathogens, such as HPV, this approach neglects possible correlation between type-specific serologic test results within subjects, caused e.g. by inter-individual differences in sexual behavior and immune response. Improvement could therefore be obtained through joint estimation of seroprevalence against multiple HPV types, as type-specific antibody concentrations often display correlation, especially when measured by multiplex immunoassays [22]. Here we propose a bivariate mixture model, in which the age-dependent HPV16 and -18 seroprevalence is jointly estimated from correlated antibody responses against HPV16 and -18. The bivariate model provides information on the joint occurrence of HPV types and is shown to enable a more reliable classification of singly and doubly seropositive individuals than a combination of separate univariate analyses.

## Methods

### Ethics statement

A signed informed consent was obtained from all participants of the serological survey carried out in the Netherlands in 2006/2007. For those below 18 years of age, signed informed consent was also obtained from the parents, guardians or care takers. The study proposal was approved by the medical ethics testing committee of the foundation of therapeutic evaluation of medicines (METC-STEG) in Almere, The Netherlands (ISRCTN 20164309).

### Serological data

We analyzed the serum concentrations of HPV16 and -18 immunoglobulin G (IgG) antibodies in women who participated in a large cross-sectional survey, representative of the Dutch general population. The samples were collected in 2006/2007, before the introduction of HPV16 and -18 vaccination in the Dutch national immunization program in 2009. A total of 3,875 randomly sampled women between 0 and 79 years of age provided a serum sample. HPV type-specific IgG antibodies against L1 virus-like particles (VLP) were tested with a VLP-based multiplex immunoassay [23]. The assay measures the antibody concentrations to 7 high-risk HPV types simultaneously, and it has a lower limit of detection at 0.08 luminex units per milliliter (LU/mL) for HPV16 and at 0.03 LU/mL for HPV18.

### Bivariate mixture model

The log-transformed HPV16 and -18 antibody concentrations $x_{i}=\left(x_{i}^{16},x_{i}^{18}\right)$ of observations *i* = 1, …, 3875 are described by a Gaussian mixture model:

$$x_{i}\;\sim \;N\left(\mu_{z_{i}},\Sigma_{z_{i}}\right)$$

with four bivariate normal component densities with unknown means and covariance matrices. The four component densities represent individuals that are:

1. Seronegative for both HPV types, with mean and covariance matrix:
  $$\mu_{1}=\left(\begin{array}{c} \mu_{--}^{16}\\ \mu_{--}^{18} \end{array}\right)\;\;\Sigma_{1}=\left(\begin{array}{cc} {\left(\sigma_{--}^{16}\right)}^{2} & \rho_{--}\sigma_{--}^{16}\sigma_{--}^{18}\\ \rho_{--}\sigma_{--}^{16}\sigma_{--}^{18} & {\left(\sigma_{--}^{18}\right)}^{2} \end{array}\right)$$
2. Seropositive for HPV16 and seronegative for HPV18, with mean and covariance matrix:
  $$\mu_{2}=\left(\begin{array}{c} \mu_{+-}^{16}\\ \mu_{+-}^{18} \end{array}\right)\;\;\Sigma_{2}=\left(\begin{array}{cc} {\left(\sigma_{+-}^{16}\right)}^{2} & \rho_{+-}\sigma_{+-}^{16}\sigma_{+-}^{18}\\ \rho_{+-}\sigma_{+-}^{16}\sigma_{+-}^{18} & {\left(\sigma_{+-}^{18}\right)}^{2} \end{array}\right)$$
3. Seropositive for HPV18 and seronegative for HPV16, with mean and covariance matrix:
  $$\mu_{3}=\left(\begin{array}{c} \mu_{-+}^{16}\\ \mu_{-+}^{18} \end{array}\right)\;\;\Sigma_{3}=\left(\begin{array}{cc} {\left(\sigma_{-+}^{16}\right)}^{2} & \rho_{-+}\sigma_{-+}^{16}\sigma_{-+}^{18}\\ \rho_{-+}\sigma_{-+}^{16}\sigma_{-+}^{18} & {\left(\sigma_{-+}^{18}\right)}^{2} \end{array}\right)$$
4. Seropositive for both HPV types, with mean and covariance matrix:
  $$\mu_{4}=\left(\begin{array}{c} \mu_{++}^{16}\\ \mu_{++}^{18} \end{array}\right)\;\;\Sigma_{4}=\left(\begin{array}{cc} {\left(\sigma_{++}^{16}\right)}^{2} & \rho_{++}\sigma_{++}^{16}\sigma_{++}^{18}\\ \rho_{++}\sigma_{++}^{16}\sigma_{++}^{18} & {\left(\sigma_{++}^{18}\right)}^{2} \end{array}\right)$$

The subscripts of the means, standard deviations, and correlations (parameters *μ*, *σ* and *ρ* respectively) indicate to which mixture component the parameter belongs (e.g., the HPV16 seropositive and HPV18 seronegative component is represented by + −), and the superscript represents the HPV type.

Furthermore, *z*_*i*_ specifies the unobserved mixture component that observation *i* originated from, which is modelled as a random latent variable:

$$z_{i}\;\sim \;\text{Categorical}\left(\phi_{A_{i}}\right)$$

With $\phi_{A_{i},k}$ denoting the probability that individual *i* in age group *A*_*i*_ belongs to mixture component *k*, $\sum_{k=1}^{4}\phi_{A_{i},k}=1$. The population was divided into 5 age groups, i.e. *A*_*i*_ ∈ {1, 2, …, 5} corresponding to (0–10], (10–20], (20–40], (40–60], and (60–80] year olds. Because a survey on sexual behavior of youth in the Netherlands showed that intercourse below age 12 is rare [24], we assume that the antibody concentrations of the youngest age group are only informative for the mean and covariance matrix of the seronegative component, i.e. ***ϕ***_1_ = (1, 0, 0, 0) for this age group.

### Parameter estimation

Parameters were estimated by Markov chain Monte Carlo (MCMC) simulation with JAGS, a program for Bayesian analysis using Gibbs sampling. Each observation contributed to the likelihood as follows:

$$f\left(x_{i};\phi_{A_{i}},\mu ,\Sigma \right)=\sum_{k=1}^{4}\phi_{A_{i},k}N\left(x_{i};\mu_{k},\Sigma_{k}\right)$$

We imputed antibody concentrations below the detection limit of the VLP-based multiplex immunoassay (S1 Text). To avoid label switching in posterior simulations, the positive mixture means were re-parameterized as:

$$\mu_{2}=\mu_{1}+\left(\begin{array}{c} \Delta_{+-}^{16}\\ \Delta_{+-}^{18} \end{array}\right),$$

$$\mu_{3}=\mu_{1}+\left(\begin{array}{c} \Delta_{-+}^{16}\\ \Delta_{-+}^{18} \end{array}\right),$$

$$\mu_{4}=\mu_{1}+\left(\begin{array}{c} \Delta_{++}^{16}\\ \Delta_{++}^{18} \end{array}\right),$$

with all Δ’ s greater than or equal to zero. By implication, we ignore the possibility that infection with HPV16 or -18 could lead to a signal below the noise of the assay in seronegative individuals.

We took normal prior distributions for the seronegative mixture means and half-normal priors for the Δ’s. Uniform prior distributions were taken for the standard deviations and the correlation parameters, and a Dirichlet prior was assumed for the mixing proportions. We ran four parallel MCMC chains. Convergence of the MCMC chains was inspected visually. Computations were performed with the statistical software R (S2 Text).

### Model scenarios and model selection

Univariate estimates of HPV16 and -18 seroprevalence (S1 Fig) are presented both discretized by age group and as a smooth function of age. We evaluate a suite of mixture models to describe the joint distribution of HPV16 and -18 antibody concentrations (Table 1). Scenario 1 assumes that HPV16 and -18 antibody concentrations are independent and follow two-component mixture distributions. The model of Scenario 1 is basically the product of two univariate mixture models, one for HPV16 and one for HPV18. Likewise, the model of Scenario 1 has twice the number of parameters of a univariate model, i.e. two μ’s and two Δ’s, four *σ*’s and eight *ϕ*’s (two per age group above 10 years).

**Table 1:** Overview of the model assumptions of five bivariate mixture models for describing the HPV16 and HPV18 antibody concentrations.

	Correlation between HPV16 and -18 antibody concentrations	Association in HPV16 and -18 seropositivity	Number of mixture components per HPV type	Number of parameters
Scenario 1	*ρ*_−−_ = *ρ*_+−_ = *ρ*_−+_ = *ρ*_++_ = 0	Independent occurrence per age group;*ϕ*_*A*_(+ +) = *ϕ*_*A*_(+ *)*ϕ*_*A*_(* +) with *ϕ*_*A*_(+ *)*ϕ*_*A*_(* +) the marginal proportions	Two marginal components; $\Delta_{++}^{16}=\Delta_{+-}^{16}\wedge \sigma_{++}^{16}=\sigma_{+-}^{16}$ and $\Delta_{++}^{18}=\Delta_{-+}^{18}\wedge \sigma_{++}^{18}=\sigma_{-+}^{18}$	16
Scenario 2	*ρ*_−−_ = *ρ*_+−_ = *ρ*_−+_ = *ρ*_++_ = 0	No restrictions above age 10 years; *ϕ*_*A*_(+ +) ≠ *ϕ*_*A*_(+ *)*ϕ*_*A*_(* +) for *A* ∈ {2,…, 5}	Two marginal components (see Scenario 1)	20
Scenario 3	No restrictions, antibody concentrations may be correlated	Independent occurrence per age group (see Scenario 1)	Two marginal components (see Scenario 1)	20
Scenario 4	No restrictions, antibody concentrations may be correlated	No restrictions above age 10 years (see Scenario 2)	Two marginal components (see Scenario 1)	24
Scenario 5	No restrictions, antibody concentrations may be correlated	No restrictions above age 10 years (see Scenario 2)	Three marginal components; $\Delta_{++}^{16}\neq \Delta_{+-}^{16}\wedge \sigma_{++}^{16}\neq \sigma_{+-}^{16}$ and $\Delta_{++}^{18}\neq \Delta_{+-}^{18}\wedge \sigma_{++}^{18}\neq \sigma_{+-}^{18}$	28

The assumption of independent occurrence in seropositivity for both types is relaxed in Scenario 2 (adding four*ϕ*’s to the model), whereas Scenario 3 allows for correlated antibody concentrations per mixture component (adding four *ρ*’s to the model). Scenario 4 allows for associations in HPV16 and -18 seropositivity as well as correlated antibody concentrations, but retains the constraint of a marginal description by two mixture components, i.e. $\Delta_{+-}^{16}=\Delta_{++}^{16}$ and $\Delta_{-+}^{18}=\Delta_{++}^{18}$. We relax this constraint in Scenario 5: the seropositive means and covariance matrices may be different for the doubly positive and singly positive mixture components, e.g. due to boosting of antibody concentrations upon multiple infections. Thus, the model of Scenario 5 has four additional parameters (two Δ’s and two *σ*’s) relative to Scenario 4, yielding a total of 28 parameters. To avoid the overfitting of assay noise in the seronegative component, which contains most of the data, we retained the assumption of constant seronegative means (i.e., $\mu_{--}^{16}=\mu_{-+}^{16},\;\mu_{--}^{18}=\mu_{+-}^{18},\;\sigma_{--}^{16}=\sigma_{-+}^{16}$ and $\sigma_{--}^{18}=\sigma_{+-}^{18}$) in all model scenarios.

Finite mixture models present some problems to the use of model selection criteria, in particular for selecting the number of mixture components [25]. We used the deviance information criterion (DIC) for model selection. The DIC balances the expected deviance—a measure of model fit—and the effective number of parameters—a measure of model complexity [26].The expected deviance was estimated by taking the average value of the deviance (defined as –2 times the log-likelihood) across posterior simulations. The effective number of parameters was computed by taking the difference between the expected deviance and the deviance at the posterior means of the parameters for the model [26]. Owing to our parameterization, we found that the posterior means adequately summarized the central tendency of the posterior densities. As an alternative, we also calculated the effective number of parameters as one half the variance across posterior simulations. This approach is invariant to re-parameterization, but assumes negligible prior information [27].

Although there is not a formal threshold to assign a relevant difference between two models, a difference of more than 7 to 10 points is generally taken to favor the model with the smaller DIC [26, 27]. To assess whether the preferred bivariate model (i.e., the model with the lowest DIC) yields a better mixture classifier than a combination of two univariate models, we simulated 50 bivariate data sets of 3800 individuals (roughly corresponding to the size of the serological survey) using the observed age distribution and parameter estimates of the selected bivariate model. Each simulated data set was fitted by both the preferred bivariate model and by two univariate models for each HPV type separately. Per data set, we calculated the probability for each individual of belonging to one of the four mixture components by Monte Carlo integration [28]. We assigned each individual to the component with the largest support, and compared how many individuals were correctly classified by the bivariate model and by the combination of two univariate models.

## Results

### Parameter estimates

**Fig 1:**
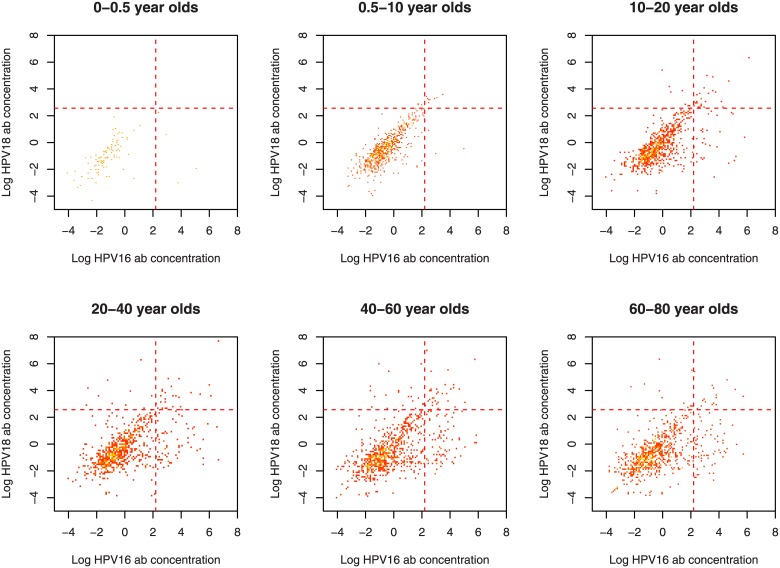
Log-transformed HPV16 and HPV18 antibody concentrations by age group. The dashed vertical line denotes the laboratory cut-off for HPV16 seropositivity and the dashed horizontal line denotes the laboratory cut-off for HPV18 seropositivity.

We observe a general increase of antibody concentrations with age, and strong correlations between the HPV16 and -18 responses across the range of antibody concentrations (Fig 1). As a result (Table 2), model scenarios that allow for correlation between the HPV16 and -18 antibody responses (Scenarios 3–5) invariably outperform the models without correlation (Scenarios 1–2). Of the models that allow for correlated antibody responses, those that also take age-dependent associations between HPV16 and -18 seropositivity into account (Scenarios 4 and 5) provide a substantially better fit than the model that ignores such associations (Scenario 3). Finally, the difference in DIC between the two remaining scenarios is large enough to favor the model with separate mixture distributions for the singly and doubly seropositive components (Scenario 5). Alternative choices for computation of the DIC had no influence on the ranking of models, as the effective number of parameters reflected expectation in most model Scenarios (Tables 1 and 2).

**Table 2:** Model information criteria of five bivariate mixture models for describing the HPV16 and HPV18 antibody concentrations. See Table 1 for explanation of scenarios. $\bar{D\left(\theta \right)}$: posterior mean deviance. $D\left(\bar{\theta }\right)$: deviance of posterior means.$p_{D}^{\left(1\right)}$: effective dimension as $\bar{D\left(\theta \right)}-D\left(\bar{\theta }\right)$ [26]. *DIC*^(1)^: deviance information criterion as $\bar{D\left(\theta \right)}+p_{D}^{\left(1\right)}$. $p_{D}^{\left(2\right)}$: effective dimension as $\frac{1}{2}\text{VAR}\left(D\left(\theta \right)\right)$ [27]. *DIC*^(2)^: deviance information criterion as $\bar{D\left(\theta \right)}+p_{D}^{\left(2\right)}$.

	$\bar{D\left(\theta \right)}$	$D\left(\bar{\theta }\right)$	$p_{D}^{\left(1\right)}$	*DIC*^(1)^	$p_{D}^{\left(2\right)}$	*DIC*^(2)^
Scenario 1	27378.3	27362.5	15.8	27394.1	17.8	27396.1
Scenario 2	26656.7	26640.5	16.2	26672.8	21.1	26677.8
Scenario 3	25077.8	25057.3	20.6	25098.4	21.0	25098.9
Scenario 4	24895.3	24873.0	22.3	24917.6	26.9	24922.3
Scenario 5	24869.0	24842.1	26.9	24896.0	28.7	24897.8

**Fig 2:**
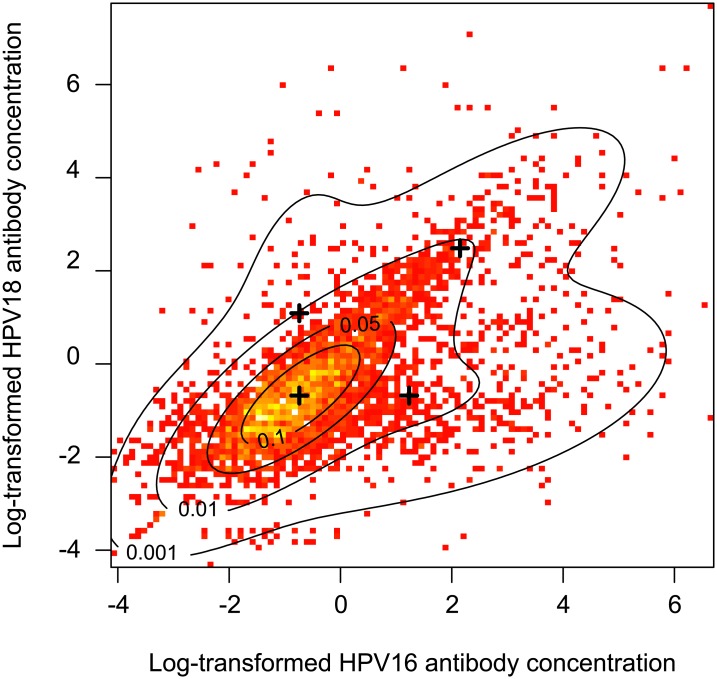
Log-transformed HPV16 and HPV18 antibody concentrations. Heat plot of log-transformed antibody concentrations of HPV16 and HPV18, with contours of the preferred bivariate mixture model (Scenario 5). Crosses denote the means of the mixture distributions.

Parameter estimates are presented in Table 3. The means of the seronegative component are substantially smaller than the means of the doubly seropositive component (-0.74 versus 2.15 for HPV16, and -0.68 versus 2.49 for HPV18). For each type, the singly seropositive means lie in between the seronegative and doubly seropositive means, as might be expected. Posterior distributions of the preferred bivariate model (Scenario 5) are summarized by median and 95% credible interval (S1 Table), and the model fit is illustrated by contour lines of the mixture density to the heat plot of the data (Fig 2). HPV16 and -18 antibody concentrations are strongly correlated in the seronegative and doubly seropositive components, with almost identical correlation on a logarithmic scale (Pearson correlation coefficients *ρ*_−−_ = 0.75 and *ρ*_++_ = 0.73, respectively). Correlation coefficients in the singly seropositive components are also comparable, but substantially smaller (*ρ*_+−_ = 0.39 and *ρ*_−+_ = 0.29).

**Table 3:** Estimated parameters (median values) of the bivariate mixture distibutions. See Table 1 for explanation of scenarios.

	HPV16- HPV18-	HPV16+ HPV18-	HPV16- HPV18+	HPV16+ HPV18+
Scenario 1	$\;N\left(\left(\begin{array}{c} -0.66\\ -0.71 \end{array}\right),\left(\begin{array}{cc} 1.15^{2} & 0\\ 0 & 1.09^{2} \end{array}\right)\right)$	$N\left(\left(\begin{array}{c} 2.49\\ -0.71 \end{array}\right),\left(\begin{array}{cc} 1.46^{2} & 0\\ 0 & 1.09^{2} \end{array}\right)\right)$	$N\left(\left(\begin{array}{c} -0.66\\ 2.12 \end{array}\right),\left(\begin{array}{cc} 1.15^{2} & 0\\ 0 & 1.58^{2} \end{array}\right)\right)$	$N\left(\left(\begin{array}{c} 2.49\\ 2.12 \end{array}\right),\left(\begin{array}{cc} 1.46^{2} & 0\\ 0 & 1.58^{2} \end{array}\right)\right)$
Scenario 2	$N\left(\left(\begin{array}{c} -0.77\\ -0.80 \end{array}\right),\left(\begin{array}{cc} 1.18^{2} & 0\\ 0 & 1.06^{2} \end{array}\right)\right)$	$N\left(\left(\begin{array}{c} 1.99\\ -0.80 \end{array}\right),\left(\begin{array}{cc} 2.37^{2} & 0\\ 0 & 1.06^{2} \end{array}\right)\right)$	$N\left(\left(\begin{array}{c} -0.77\\ 1.53 \end{array}\right),\left(\begin{array}{cc} 1.18^{2} & 0\\ 0 & 2.65^{2} \end{array}\right)\right)$	$N\left(\left(\begin{array}{c} 1.99\\ 1.53 \end{array}\right),\left(\begin{array}{cc} 2.37^{2} & 0\\ 0 & 2.65^{2} \end{array}\right)\right)$
Scenario 3	$N\left(\left(\begin{array}{c} -0.65\\ -0.57 \end{array}\right),\left(\begin{array}{cc} 1.16^{2} & 1.12\\ 1.12 & 1.21^{2} \end{array}\right)\right)$	$N\left(\left(\begin{array}{c} 1.09\\ -0.57 \end{array}\right),\left(\begin{array}{cc} 2.10^{2} & 1.11\\ 1.11 & 1.21^{2} \end{array}\right)\right)$	$N\left(\left(\begin{array}{c} -0.65\\ 1.34 \end{array}\right),\left(\begin{array}{cc} 1.16^{2} & 0.96\\ 0.96 & 2.49^{2} \end{array}\right)\right)$	$N\left(\left(\begin{array}{c} 1.09\\ 1.34 \end{array}\right),\left(\begin{array}{cc} 2.10^{2} & 4.52\\ 4.52 & 2.49^{2} \end{array}\right)\right)$
Scenario 4	$N\left(\left(\begin{array}{c} -0.75\\ -0.69 \end{array}\right),\left(\begin{array}{cc} 1.10^{2} & 0.90\\ 0.90 & 1.11^{2} \end{array}\right)\right)$	$N\left(\left(\begin{array}{c} 1.62\\ -0.69 \end{array}\right),\left(\begin{array}{cc} 1.87^{2} & 0.91\\ 0.91 & 1.11^{2} \end{array}\right)\right)$	$N\left(\left(\begin{array}{c} -0.75\\ 1.97 \end{array}\right),\left(\begin{array}{cc} 1.10^{2} & 0.69\\ 0.69 & 1.74^{2} \end{array}\right)\right)$	$N\left(\left(\begin{array}{c} 1.62\\ 1.97 \end{array}\right),\left(\begin{array}{cc} 1.87^{2} & 2.86\\ 2.86 & 1.74^{2} \end{array}\right)\right)$
Scenario 5	$N\left(\left(\begin{array}{c} -0.74\\ -0.68 \end{array}\right),\left(\begin{array}{cc} 1.09^{2} & 0.89\\ 0.89 & 1.10^{2} \end{array}\right)\right)$	$N\left(\left(\begin{array}{c} 1.23\\ -0.68 \end{array}\right),\left(\begin{array}{cc} 2.07^{2} & 0.90\\ 0.90 & 1.10^{2} \end{array}\right)\right)$	$N\left(\left(\begin{array}{c} -0.74\\ 1.09 \end{array}\right),\left(\begin{array}{cc} 1.09^{2} & 0.82\\ 0.82 & 2.59^{2} \end{array}\right)\right)$	$N\left(\left(\begin{array}{c} 2.15\\ 2.49 \end{array}\right),\left(\begin{array}{cc} 1.42^{2} & 1.28\\ 1.28 & 1.24^{2} \end{array}\right)\right)$

### Seroprevalence by age

**Fig 3:**
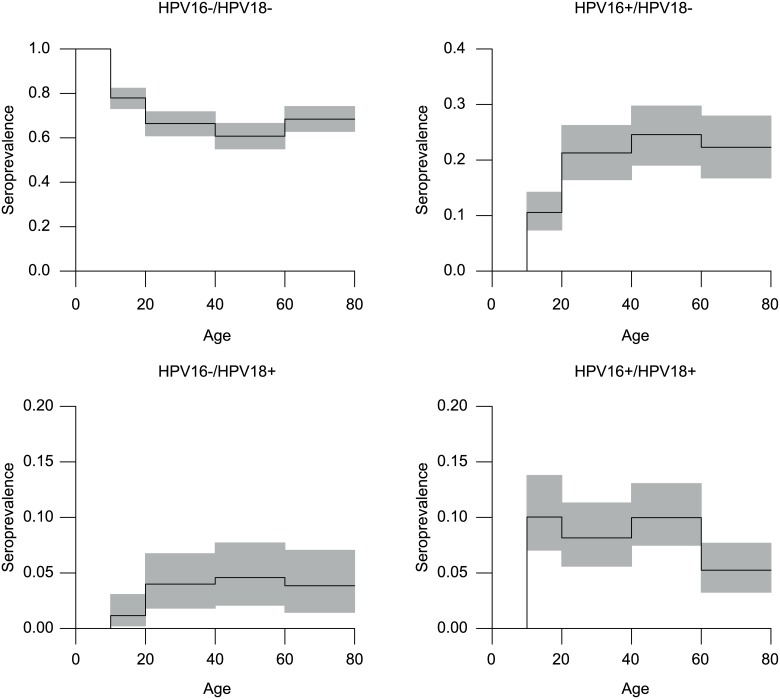
Estimated age-specific seroprevalence per mixture component. The figures represent the estimated seroprevalences for the model that allows for correlated HPV16 and -18 antibody concentrations, age-dependent associations in HPV16 and -18 seropositivity, and for three marginal mixture components per HPV type (Scenario 5). The solid lines are the median values, the shaded area represent the 95% credible interval.

Fig 3 shows the estimated age-dependent seroprevalences. Seronegativity for both types decreases from 100% in young children to approximately 60% in persons aged 40 years and older. Singly seropositive prevalence increases quickly from 10 years onwards to more than 20% for HPV16 and to 3–4% for HPV18. Seropositivity for both types also increases quickly with age, to 8–10% in 10–60 years old persons. There is a strong association in seroprevalence for HPV types 16 and 18 as demonstrated by the odds ratio (OR) for double seropositivity; we consider these as age-dependent 2-by-2 contingency tables obtained from the age-dependent mixing proportions. The association of HPV16 and HPV18 is especially pronounced for the age group 10–20 years (OR = 64), and decreases in older age groups: OR = 6.4 for 20–40 year-olds, OR = 5.4 for 40–60 year-olds, and OR = 4.2 for the oldest age group (Table 4). Note that the association in HPV16 and -18 seroprevalence is significant in all age groups.

**Table 4:** Age-specific odds ratio (95% credible interval) for the association of HPV16 and HPV18 seropositivity.

Age group (years)	0–10	10–20	20–40	40–60	60–80
Odds ratio	NA	64.3 (21.4–356)	6.4 (3.2–15.0)	5.4 (2.8–12.8)	4.2 (1.8–12.0)

**Fig 4:**
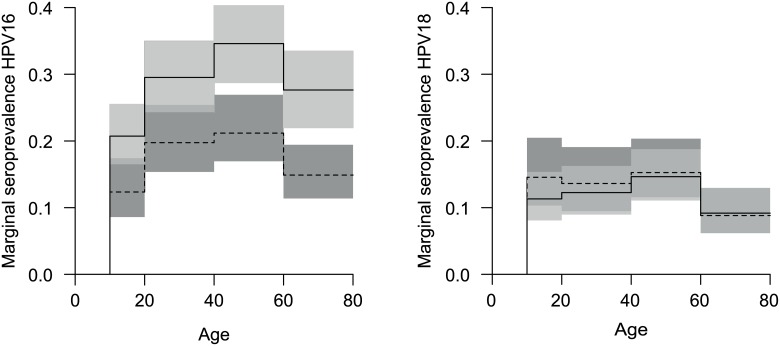
Marginal HPV16 and HPV18 seroprevalence in women. The dashed lines denote the seroprevalence from the univariate mixture model, the solid lines are from the preferred bivariate mixture model (Scenario 5). The lines are the median values, the shaded areas represent the 95% credible interval.

Both the component densities and the marginal seroprevalences from Scenario 1 are similar to the estimates from the type-specific univariate mixture models (S1 Fig). The estimated marginal seroprevalence of HPV16 is substantially higher in the preferred bivariate model than in the univariate HPV16 model (Fig 4). The difference in seroprevalence is largest for the age group 40–60 years, with an absolute difference of 13.5% in point estimates. Despite this difference, the marginal fits to the data are quite similar for the univariate and bivariate mixture models (S2 Fig). Differences between the univariate and bivariate mixture models are generally small (<2%) regarding the estimated HPV18 seroprevalence.

### Classification accuracy

The bivariate model provided better classification of individuals than a combination of univariate models for HPV16 and HPV18 in 49 out of 50 simulated data sets. The two approaches perform equally well in classifying the doubly seropositive persons: respectively 85.7% and 85.3% of these are correctly classified in a bivariate or a combined univariate analysis. However, the bivariate model provides a better classification in all other situations, in particular when classification of the singly seropositive persons is concerned: 64.3% versus 52.1% of singly HPV16 seropositive individuals and 58.9% versus 50.4% of singly HPV18 seropositive individuals are correctly classified in a bivariate versus a combined univariate analysis (Table 5).

**Table 5:** Contingency table of actual and predicted serological status of 3800 simulated individuals using parameter estimates of model Scenario 5, classified by the preferred bivariate model or by two univariate models for each HPV type separately.

Actual serostatus		Predicted serostatus				
	N#		HPV16- HPV18-	HPV16+ HPV18-	HPV16- HPV18+	HPV16+ HPV18+
HPV16- HPV18-	2695	Bivariate	**2167.72**	193.62	147.08	186.58
		Univariate	**2148.52**	175.88	192.58	178.02
HPV16+ HPV18-	637	Bivariate	169.76	**409.88**	26.48	30.88
		Univariate	216.62	**331.78**	16.04	72.56
HPV16- HPV18+	168	Bivariate	47.08	9.52	**98.90**	12.50
		Univariate	61.04	5.22	**84.70**	17.04
HPV16+ HPV18+	297	Bivariate	17.46	7.08	17.82	**254.64**
		Univariate	10.46	6.06	27.06	**253.42**

^#^Numbers are averaged over 50 simulations; bold figures denote correctly classified cases.

## Discussion

We have presented a bivariate mixture model for joint estimation of seroprevalence against multiple genotypes in seroepidemiologic studies. The method is particularly useful when type-specific antibody measurements are correlated, a typical observation when using multiplex immunoassay technology. We applied our model to estimate HPV16 and -18 seroprevalence in women prior to introducing HPV vaccination of preadolescent girls in the Netherlands. Our results provide valuable baseline estimates of vaccine-type seroprevalence that can be used for future vaccine impact assessment.

Our method provides a full description of the HPV16 and -18 antibody concentrations, and allows for post-hoc assessment of age-dependent associations in HVP16 and -18 seropositivity. Our analysis has uncovered that (*i*) antibody concentrations in seronegative and doubly seropositive individuals are strongly correlated; (*ii*) doubly seropositive individuals have higher antibody concentrations than singly seropositive individuals; and (*iii*) there is a positive association between HPV16 and -18 seropositivity in all age groups, with the strongest association around the age of sexual debut.

Age-dependent co-occurrence in cross-sectional serological data has been dealt with earlier using methods that rely on marginal models for multivariate binary data, i.e. denoting seropositivity for each infection separately. These methods model the odds ratio of co-occurrence either by means of a parametric link function as in the bivariate Dale model [29, 30] or via correlated frailty models [31, 32], and are based on a rigid classification of sera using a predefined cut-off value. However, in the seroepidemiology of some infections, such as HPV, considerable uncertainty exists about the true serological status of individual samples, and population prevalence needs to be inferred from an analysis that takes account of this uncertainty. In addition, the choice of a link function or frailty distribution is not straightforward if the odds ratio is strongly peaked as a function of age, which we show to be the case for HPV16 and -18 seropositivity. Our bivariate mixture model circumvents this problem and gives an unconstrained estimate of co-occurrence of antibodies against multiple infections.

Our analysis was stratified into age groups of 10 to 20 years to account for changes in seroprevalence and co-occurrence by age. We showed for the univariate analyses that the stratified seroprevalence resembled the smoothed seroprevalence figures. A straightforward multivariate extension of the smoothing spline approach would involve modeling the mixing proportions by a flexible but age-dependent function, for example by means of vector generalized additive models [33]. This would be especially worthwhile for applications to smaller data sets or with an uneven distribution of serum samples across age groups. Such an extension would also yield a better resolution with respect to the age-specific pattern of co-occurrence between HPV16 and HPV18 seropositivity.

Positive associations between HPV type-specific seropositivity are to be expected, as persons who have been infected with one type are at increased risk for acquisition of a second type [34]. This has already been confirmed by previous analyses based on serological data [35–38], but to our knowledge we are the first to uncover and estimate age dependency in the association of HPV16 and -18 seropositivity. Our analyses show that this association is particularly strong around the age of sexual debut. This suggests that the population of adolescents and young adults is strongly heterogeneous with respect to sexual activity, which is in line with survey data on sexual behavior. The substantially smaller odds ratios among 20–40 year-olds can possibly be explained by the fact that almost 90% of women have had sexual intercourse by the age of 21 [24]. Heterogeneity with respect to lifetime number of sex partners may explain the clustering of HPV16 and -18 seropositivity at older age, as the association remains significant in all age groups. Nevertheless, the decreasing odds ratios with age suggests that older age groups are more homogeneous with regard to HPV16 and -18 infection history, presumably because both types are relatively common in sexually active age groups and are easily transmitted within sexual partnerships [39].

We have estimated the correlation between HPV16 and -18 antibody concentrations for each of the mixture components separately. The correlation among seronegative individuals may represent assay noise, given that seronegativity is related to absence of prior exposure to HPV16 and -18, certainly at young age. As we expect that doubly seropositive persons have experienced both HPV16 and -18 infections, the correlation in the doubly seropositive component could also reflect the immunologic profile of a host’s response to infection; a strong antibody response to HPV16 is likely related to a strong antibody response to HPV18. Additionally, we found that the antibody concentrations of the doubly seropositive individuals were larger than those measured for the same HPV type in singly seropositive individuals. Possibly, the assay targets not only antibodies to the main L1 epitope but also recognizes epitopes that are shared between HPV16 and HPV18 [40], which would result in a boosted immune response to one type after infection with the other type.

The estimated HPV16 seroprevalence is substantially larger in bivariate than in univariate analysis, even though both analyses achieved comparable marginal fits to the data. Using a simulation study, we concluded that the bivariate mixture model is better able to correctly classify seropositive individuals, as it enables more flexibility in describing antibody concentrations among HPV16 seropositives by stratification for HPV18 serostatus. Indeed, the increased HPV16 seroprevalence in the bivariate model was predominantly due to reclassification of samples with HPV16-specific antibody concentrations that had similar support for seronegativity or seropositivity in the univariate analysis. This is relevant for post-vaccine serological surveys, because a reduced circulation of vaccine-type HPV throughout the population is not only expected to lower the proportion seropositives among the non-vaccinated individuals, but also the relative rate of double seropositivity against HPV16 and -18. Vaccination will probably change the association between HPV16 and -18 seropositivity, in turn leading to varying degrees of seroprevalence underestimation by univariate analyses in pre- and post-vaccine serological surveys. A bivariate mixture model would likely provide more sensitive and accurate estimates of changing seroprevalence by age in partly vaccinated cohorts. Our results could therefore be particularly useful for the assessment of population effects from HPV vaccination in forthcoming serological surveys.

## Supporting Information

S1 Fig Univariate mixture model estimates of HPV16 and HPV18 seroprevalence in women. Seroprevalence is either discretized in five age groups (step function) or modeled by a P-spline (smoothed line) according to ref (16).
(EPS)

S2 Fig Marginal distribution of HPV16 antibody concentrations by age group. Log-transformed HPV16 antibody concentrations of the serological data are plotted in a histogram, together with the estimated marginal density of the univariate mixture model (grey line) and the preferred bivariate mixture model (black line).
(EPS)

S1 Table Parameter estimates of the preferred bivariate model. (DOC)

S1 Text Imputation of observations below detection limit of the multiplex immunoassay. (DOC)

S2 Text R code of the bivariate mixture model. (DOC)

## References

[1] Bosch FX, Broker TR, Forman D, Moscicki AB, Gillison ML, Doorbar J, et al Comprehensive control of human papillomavirus infections and related diseases. Vaccine. 2013;31(Suppl 8): I1–I31. 10.1016/j.vaccine.2013.07.026 24229716 PMC4062073

[2] Li N, Franceschi S, Howell-Jones R, Snijders PJ, Clifford GM. Human papillomavirus type distribution in 30,848 invasive cervical cancers worldwide: Variation by geographical region, histological type and year of publication. Int J Cancer. 2011;128: 927–935. 10.1002/ijc.25396 20473886

[3] De Vuyst H, Clifford GM, Nascimento MC, Madeleine MM, Franceschi S. Prevalence and type distribution of human papillomavirus in carcinoma and intraepithelial neoplasia of the vulva, vagina and anus: a meta-analysis. Int J Cancer. 2009; 124: 1626–1636. 10.1002/ijc.24116 19115209

[4] Miralles-Guri C, Bruni L, Cubilla AL, Castellsagué X, Bosch FX, de Sanjosé S. Human papillomavirus prevalence and type distribution in penile carcinoma. J Clin Pathol. 2009;62: 870–878. 10.1136/jcp.2008.063149 19706632

[5] Abogunrin S, Di Tanna GL, Keeping S, Carroll S, Iheanacho I. Prevalence of human papillomavirus in head and neck cancers in European populations: a meta-analysis. BMC Cancer. 2014;14: 968 10.1186/1471-2407-14-968 25515630 PMC4320477

[6] Schurink-van ‘t Klooster TM, de Melker HE, editors. The National Immunisation Programme in the Netherlands: Surveillance and Developments in 2013–2014. Bilthoven: National Institute for Public Health and the Environment; 2014.

[7] Vink MA, Bogaards JA, van Kemenade FJ, de Melker HE, Meijer CJ, Berkhof J. Clinical progression of high-grade cervical intraepithelial neoplasia: estimating the time to preclinical cervical cancer from doubly censored national registry data. Am J Epidemiol. 2013;178: 1161–1169. 10.1093/aje/kwt077 23897645

[8] Hariri S, Markowitz LE, Bennett NM, Niccolai LM, Schafer S, Bloch K, et al Monitoring effect of human papillomavirus vaccines in US population, emerging infections program, 2008–2012. Emerg Infect Dis. 2015;21: 1557–1561. 10.3201/eid2109.141841 26291379 PMC4550135

[9] Elfström KM, Dillner J, Arnheim-Dahlström L. Organization and quality of HPV vaccination programs in Europe. Vaccine. 2015;33: 1673–1681. 10.1016/j.vaccine.2015.02.028 25720792

[10] Farrington CP, Kanaan MN, Gay NJ. Estimation of the basic reproduction number for infectious diseases from age-stratified serological survey data (with discussion). J R Stat Soc Ser C Appl Stat. 2001;50: 251–292.

[11] Hens N, Shkedy Z, Aerts M. Modeling infectious disease parameters based on serological and social contact data: a modern statistical perspective. 1st ed New York: Springer; 2012.

[12] Drolet M, Bénard É, Boily MC, Ali H, Baandrup L, Bauer H, et al Population-level impact and herd effects following human papillomavirus vaccination programmes: a systematic review and meta-analysis. Lancet Infect Dis. 2015;15: 565–580. 10.1016/S1473-3099(14)71073-4 25744474 PMC5144106

[13] Brisson M, Van de Velde N, Boily MC. Different population-level vaccination effectiveness for HPV types 16, 18, 6 and 11. Sex Transm Infect. 2011;87: 41–43. 10.1136/sti.2010.044412 20924049

[14] Bogaards JA, Coupé VM, Xiridou M, Meijer CJ, Wallinga J, Berkhof J. Long-term impact of human papillomavirus vaccination on infection rates, cervical abnormalities, and cancer incidence. Epidemiology. 2011;22: 505–515. Erratum in: Epidemiology. 2011;22: 881. 10.1097/EDE.0b013e31821d107b 21540743

[15] Bogaards JA, Wallinga J, Brakenhoff RH, Meijer CJ, Berkhof J. Direct benefit of vaccinating boys along with girls against oncogenic human papillomavirus: Bayesian evidence synthesis. BMJ. 2015;350: h2016 10.1136/bmj.h2016 25985328 PMC4428278

[16] Schiffman M, Boyle S, Raine-Bennett T, Katki HA, Gage JC, Wentzensen N, et al The role of human papillomavirus genotyping in cervical cancer screening: a large-scale evaluation of the cobas HPV test. Cancer Epidemiol Biomarkers Prev. 2015;24: 1304–1310. 10.1158/1055-9965.EPI-14-1353 26088703 PMC4560647

[17] Smits G, Mollema L, Hahné S, de Melker H, Tcherniaeva I, Waaijenborg S, et al Seroprevalence of mumps in The Netherlands: dynamics over a decade with high vaccination coverage and recent outbreaks. PLoS One. 2013;8: e58234 10.1371/journal.pone.0058234 23520497 PMC3592917

[18] Smits G, Mollema L, Hahné S, de Melker H, Tcherniaeva I, van der Klis F, et al Seroprevalence of rubella antibodies in The Netherlands after 32 years of high vaccination coverage. Vaccine. 2014;32: 1890–1895. 10.1016/j.vaccine.2014.01.066 24513012

[19] Schwarz TF, Leo O. Immune response to human papillomavirus after prophylactic vaccination with AS04-adjuvanted HPV-16/18 vaccine: improving upon nature. Gynecol Oncol. 2008;110(3 Suppl 1): S1–S10. 10.1016/j.ygyno.2008.05.036 18653222

[20] Stanley M. Immunobiology of HPV and HPV vaccines. Gynecol Oncol 2008;109(2 Suppl): S15–S21. 10.1016/j.ygyno.2008.02.003 18474288

[21] Vink MA, van de Kassteele J, Wallinga J, Teunis PF, Bogaards JA. Estimating seroprevalence of human papillomavirus type 16 using a mixture model with smoothed age-dependent mixing proportions. Epidemiology. 2015;26: 8–16. 10.1097/EDE.0000000000000196 25380503

[22] de Koning L, Liptak C, Shkreta A, Bradwin G, Hu FB, Pradhan AD, et al A multiplex immunoassay gives different results than singleplex immunoassays which may bias epidemiologic associations. Clin Biochem. 2012;45: 848–851. 10.1016/j.clinbiochem.2012.04.006 22537455

[23] Scherpenisse M, Mollers M, Schepp RM, Boot HJ, de Melker HE, Meijer CJ, et al Seroprevalence of seven high-risk HPV types in The Netherlands. Vaccine. 2012;30: 6686–6693. 10.1016/j.vaccine.2012.08.068 22959981

[24] de Graaf H, Meijer S, Poelman J, Vanwesenbeeck I. Sex Below the Age of 25: Sexual Health of Youth in the Netherlands in 2005 [in Dutch]. Delft: Eburon Academic Publishers:; 2005.

[25] Plummer M. Penalized loss functions for Bayesian model comparison. Biostatistics. 2008;9: 523–539. 10.1093/biostatistics/kxm049 18209015

[26] Spiegelhalter DJ, Best NG, Carlin BP, van der Linde A. Bayesian measures of model complexity and fit. J R Stat Soc Series B Stat Methodol. 2002;64: 583–639.

[27] Gelman A, Carlin JB, Stern HS, Rubin DB. Bayesian Data Analysis. 2nd ed Boca Raton, FL: Chapman & Hall; 2004.

[28] Gelfand AE, Smith AF. Sampling-based approaches to calculating marginal densities. J Am Stat Assoc. 1990;85: 398–409.

[29] Dale JR. Global cross-ratio models for bivariate, discrete, ordered responses. Biometrics. 1986;42: 909–917. 3814731

[30] Hens N, Aerts M, Shkedy Z, Theeten H, Van Damme P, Beutels P. Modelling multisera data: the estimation of new joint and conditional epidemiological parameters. Stat Med. 2008;27: 2651–2664. 17972342 10.1002/sim.3089

[31] Hens N, Wienke A, Aerts M, Molenberghs G. The correlated and shared gamma frailty model for bivariate current status data: an illustration for cross-sectional serological data. Stat Med. 2009;28: 2785–2800. 10.1002/sim.3660 19591117

[32] Farrington CP, Whitaker HJ, Unkel S, Pebody R. Correlated infections: quantifying individual heterogeneity in the spread of infectious diseases. Am J Epidemiol 2013;177: 474–486. 10.1093/aje/kws260 23403987

[33] Yee TW, Wild CJ. Vector generalized additive models. J R Stat Soc Series B Stat Methodol. 1996;58: 481–493.

[34] Vaccarella S, Franceschi S, Snijders PJ, Herrero R, Meijer CJ, Plummer M. Concurrent infection with multiple human papillomavirus types: pooled analysis of the IARC HPV Prevalence Surveys. Cancer Epidemiol Biomarkers Prev. 2010;19: 503–510. 10.1158/1055-9965.EPI-09-0983 20142247

[35] Vaccarella S, Franceschi S, Clifford GM, Touzé A, Hsu CC, de Sanjosé S, et al Seroprevalence of antibodies against human papillomavirus (HPV) types 16 and 18 in four continents: the International Agency for Research on Cancer HPV Prevalence Surveys. Cancer Epidemiol Biomarkers Prev. 2010;19: 2379–2388. 10.1158/1055-9965.EPI-10-0336 20826835

[36] Combes JD, Pawlita M, Waterboer T, Hammouda D, Rajkumar T, Vanhems P, et al Antibodies against high-risk human papillomavirus proteins as markers for invasive cervical cancer. Int J Cancer. 2014;135: 2453–2461. 10.1002/ijc.28888 24729277

[37] Mooij SH, van der Klis FR, van der Sande MA, Schepp RM, Speksnijder AG, Bogaards JA, et al Seroepidemiology of high-risk HPV in HIV-negative and HIV-infected MSM: the H2M study. Cancer Epidemiol Biomarkers Prev. 2013;22: 1698–1708. 10.1158/1055-9965.EPI-13-0460 24097197

[38] Vriend HJ, Bogaards JA, van der Klis FR, Scherpenisse M, Boot HJ, King AJ, et al Patterns of human papillomavirus DNA and antibody positivity in young males and females, suggesting a site-specific natural course of infection. PLoS One. 2013;8: e60696 10.1371/journal.pone.0060696 23637760 PMC3634056

[39] Bogaards JA, Xiridou M, Coupé VM, Meijer CJ, Wallinga J, Berkhof J. Model-based estimation of viral transmissibility and infection-induced resistance from the age-dependent prevalence of infection for 14 high-risk types of human papillomavirus. Am J Epidemiol. 2010;171: 817–825. 10.1093/aje/kwp466 20231211

[40] Scherpenisse M, Schepp RM, Mollers M, Meijer CJ, Berbers GA, van der Klis FR. Characteristics of HPV-specific antibody responses induced by infection and vaccination: cross-reactivity, neutralizing activity, avidity and IgG subclasses. PLoS One. 2013;8: e74797 10.1371/journal.pone.0074797 24058629 PMC3776846

