# Supplementary material for: A Bivariate Mixture Model for Natural Antibody Levels to Human Papillomavirus Types 16 and 18: Baseline Estimates for Monitoring the Herd Effects of Immunization
Source: PLoS One. 2016 Aug 18;11(8):e0161109. doi: 10.1371/journal.pone.0161109 (PMC4990197; doi:10.1371/journal.pone.0161109)
Supplement: S1 Table — (DOC) [file pone.0161109.s003.doc]

Table S1. Estimated parameters for Scenario 5 (median and 95% credible interval).

|  | HPV16- HPV18- | | HPV16+ HPV18- | | HPV16- HPV18+ | | HPV16+ HPV18+ | |
| --- | --- | --- | --- | --- | --- | --- | --- | --- |
|  | Median | 95% CI | Median | 95% CI | Median | 95% CI | Median | 95% CI |
| 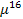 | -0.74 | (-0.79, -0.70) | 1.23 | (0.93, 1.72) | -0.74 | (-0.79, -0.70) | 2.15 | (1.78, 2.48) |
| 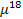 | -0.68 | (-0.72, -0.64) | -0.68 | (-0.72, -0.64) | 1.09 | (0.28, 3.15) | 2.49 | (2.14, 2.78) |
| 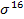 | 1.09 | (1.05, 1.21) | 2.07 | (1.86, 2.23) | 1.09 | (1.05, 1.21) | 1.42 | (1.25, 1.62) |
| 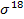 | 1.10 | (1.06, 1.13) | 1.10 | (1.06, 1.13) | 2.59 | (1.70, 3.08) | 1.24 | (1.08, 1.45) |
| 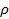 | 0.75 | (0.73, 0.77) | 0.39 | (0.30, 0.48) | 0.29 | (0.07, 0.52) | 0.73 | (0.58, 0.83) |
