# Supplementary material for: A Bivariate Mixture Model for Natural Antibody Levels to Human Papillomavirus Types 16 and 18: Baseline Estimates for Monitoring the Herd Effects of Immunization
Source: PLoS One. 2016 Aug 18;11(8):e0161109. doi: 10.1371/journal.pone.0161109 (PMC4990197; doi:10.1371/journal.pone.0161109)
Supplement: S1 Text — (DOC) [file pone.0161109.s004.doc]

**A bivariate mixture model for natural antibody levels to HPV16 and -18: baseline estimates for monitoring the herd effects of immunization**

Text S1 ***Imputation of censored observations***

We imputed antibody concentrations below the detection limit of the VLP-based multiplex immunoassay. Twenty women (0.5%) had concentrations below the detection limit for both HPV types, another 156 women (4%) only had a HPV16 concentration below detection limit, and 32 women (0.8%) only had a HPV18 concentration below detection limit.

For samples with two censored observations, we drew HPV16 and -18 log-transformed antibody concentrations below detection limit of the assay from the tail of a bivariate normal distribution, using the pmvnorm function from the mvtnorm package in R, assuming:


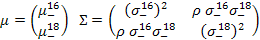


Here,
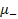
 and
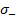
 are the estimated means and standard deviations of the seronegative component densities from the univariate two-component mixture models for HPV16 and -18 separately, and
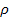
 is the correlation in the data excluding individuals with a censored observation.

For samples with only a censored HPV16 observation, we drew a point below the HPV16 detection limit from the conditional normal distribution. In case the corresponding HPV18 concentration was more likely to come from the seronegative component than from the seropositive component, the conditional mean was taken to be:


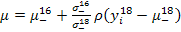


Otherwise, i.e. in case the HPV18 concentration was more likely to come from the seropositive component, the conditional mean was taken to be:


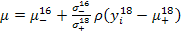


Here,
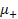
 and
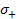
 are the estimated mean and standard deviation of the seropositive component density from the univariate two-component mixture model. In either case, the variance for imputation was taken to be
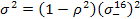
. Censored HPV18 observations were imputed analogously, i.e. if the corresponding HPV16 concentration was most likely to come from the seronegative component, we took the conditional mean to be:


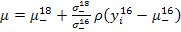


If instead the HPV18 concentration was most likely to come from the seropositive component, we took the conditional mean to be:


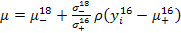


In both cases, the variance for imputation was taken to be
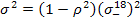
.
