# Supplementary material for: A Bivariate Mixture Model for Natural Antibody Levels to Human Papillomavirus Types 16 and 18: Baseline Estimates for Monitoring the Herd Effects of Immunization
Source: PLoS One. 2016 Aug 18;11(8):e0161109. doi: 10.1371/journal.pone.0161109 (PMC4990197; doi:10.1371/journal.pone.0161109)
Supplement: S2 Text — (DOC) [file pone.0161109.s005.doc]

**A bivariate mixture model for natural antibody levels to HPV16 and -18: baseline estimates for monitoring the herd effects of immunization**

Text S2 ***R Code Bivariate Mixture Model***

#

# Simulated data

#

library(mvtnorm)

#mu's

mu16neg.sim <- -1

mu18neg.sim <- -1

mu00.sim <- c(mu16neg.sim, mu18neg.sim)

mu10.sim <- c(4, mu18neg.sim)

mu01.sim <- c(mu16neg.sim, 4)

mu11.sim <- c(6, 6)

#sigma's

sd16neg.sim <- 1.2

sd18neg.sim <- 1.2

rho00.sim <- 0.7

rho10.sim <- 0.3

rho01.sim <- 0.3

rho11.sim <- 0.7

sd10_16.sim <- 1.1

sd01_18.sim <- 1.1

sd11_16.sim <- 1.4

sd11_18.sim <- 1.4

s00.sim <- matrix(c(sd16neg.sim^2,sd16neg.sim*sd18neg.sim*rho00.sim,sd16neg.sim*sd18neg.sim*rho00.sim,sd18neg.sim^2), nrow = 2)

s10.sim <- matrix(c(sd10_16.sim^2,sd10_16.sim*sd18neg.sim*rho10.sim,sd10_16.sim*sd18neg.sim*rho10.sim,sd18neg.sim^2), nrow = 2)

s01.sim <- matrix(c(sd16neg.sim^2,sd16neg.sim*sd01_18.sim*rho01.sim,sd16neg.sim*sd01_18.sim*rho01.sim,sd01_18.sim^2), nrow = 2)

s11.sim <- matrix(c(sd11_16.sim^2,sd11_16.sim*sd11_18.sim*rho11.sim,sd11_16.sim*sd11_18.sim*rho11.sim,sd11_18.sim^2), nrow = 2)

#age specific proportion belonging to component --, +-, -+, ++

age0 <- c(1, 0, 0, 0)

age1 <- c(0.5, 0.2, 0.1, 0.2)

age2 <- c(0.5, 0.2, 0.2, 0.1)

age3 <- c(0.3, 0.25, 0.25, 0.2)

age4 <- c(0.3, 0.25, 0.25, 0.2)

#Number of individuals belonging to age group 0,1,2,3,4

N0 <- 200

N1 <- 250

N2 <- 400

N3 <- 400

N4 <- 400

x0 <- rmvnorm(N0, mean = mu00.sim, sigma = s00.sim)

x1 <- rbind(rmvnorm(N1*age1[1], mean = mu00.sim, sigma = s00.sim),

rmvnorm(N1*age1[2], mean = mu10.sim, sigma = s10.sim),

rmvnorm(N1*age1[3], mean = mu01.sim, sigma = s01.sim),

rmvnorm(N1*age1[4], mean = mu11.sim, sigma = s11.sim))

x2 <- rbind(rmvnorm(N2*age2[1], mean = mu00.sim, sigma = s00.sim),

rmvnorm(N2*age2[2], mean = mu10.sim, sigma = s10.sim),

rmvnorm(N2*age2[3], mean = mu01.sim, sigma = s01.sim),

rmvnorm(N2*age2[4], mean = mu11.sim, sigma = s11.sim))

x3 <- rbind(rmvnorm(N3*age3[1], mean = mu00.sim, sigma = s00.sim),

rmvnorm(N3*age3[2], mean = mu10.sim, sigma = s10.sim),

rmvnorm(N3*age3[3], mean = mu01.sim, sigma = s01.sim),

rmvnorm(N3*age3[4], mean = mu11.sim, sigma = s11.sim))

x4 <- rbind(rmvnorm(N4*age4[1], mean = mu00.sim, sigma = s00.sim),

rmvnorm(N4*age4[2], mean = mu10.sim, sigma = s10.sim),

rmvnorm(N4*age4[3], mean = mu01.sim, sigma = s01.sim),

rmvnorm(N4*age4[4], mean = mu11.sim, sigma = s11.sim))

sim.data <- rbind(cbind(x0, 1), cbind(x1, 2), cbind(x2, 3), cbind(x3, 4), cbind(x4, 5))

sim.data <- as.data.frame(sim.data)

names(sim.data) <- c("log.hpv16", "log.hpv18", "Age_group")

# Plot

with(sim.data, plot(log.hpv16, log.hpv18, pch = "+"))

###################################################################################

#

# setup bivariate mixture model scenario 5

#

# working directory

setwd("/s-schijf/vinka/Pienter/Bivariate mixture")

# packages

library(parallel)

library(rjags)

# Start cluster

n.cores <- detectCores()

cl <- makeCluster(n.cores)

# JAGS function for parallel computing

runjags.par <- function(x, workdir, data, inits, n.adapt, n.burnin, n.iter, thin, variable.names) {

library(rjags)

# create JAGS model

jags.mod <- jags.model(file = file.path(workdir, "model.txt"), data = data, inits = inits, n.chains = 1, n.adapt = n.adapt, quiet = TRUE)

# burn-in

update(jags.mod, n.iter = n.burnin)

# sample from posterior

coda.samples(model = jags.mod, n.iter = n.iter, thin = thin, variable.names = variable.names)

}

#

# fit bivariate mixture model

#

# Model

model.string <- "model {

# Likelihood

for (i in 1:n) {

y[i, 1:2] ~ dmnorm(Mu[I[i], 1:2], Omega[I[i], 1:2, 1:2])

I[i] ~ dcat(pi[y[i, 3], 1:4])

}

# Precision matrices are inverse of covariances matrices

for (k in 1:4) {

Omega[k, 1:2, 1:2] <- inverse(Sigma[k, 1:2, 1:2])

}

# Fill in the parameters

# Quadrant 1: y[1] negative, y[2] negative

Mu[1, 1] <- mu[1]

Mu[1, 2] <- mu[3]

Sigma[1, 1, 1] <- sigma[1]^2

Sigma[1, 1, 2] <- sigma[1]*sigma[3]*rho[1]

Sigma[1, 2, 1] <- sigma[3]*sigma[1]*rho[1]

Sigma[1, 2, 2] <- sigma[3]^2

# Quadrant 2: y[1] positive, y[2] negative

Mu[2, 1] <- mu[2]

Mu[2, 2] <- mu[3]

Sigma[2, 1, 1] <- sigma[2]^2

Sigma[2, 1, 2] <- sigma[2]*sigma[3]*rho[2]

Sigma[2, 2, 1] <- sigma[3]*sigma[2]*rho[2]

Sigma[2, 2, 2] <- sigma[3]^2

# Quadrant 3: y[1] negative, y[2] positive

Mu[3, 1] <- mu[1]

Mu[3, 2] <- mu[4]

Sigma[3, 1, 1] <- sigma[1]^2

Sigma[3, 1, 2] <- sigma[1]*sigma[4]*rho[3]

Sigma[3, 2, 1] <- sigma[4]*sigma[1]*rho[3]

Sigma[3, 2, 2] <- sigma[4]^2

# Quadrant 4: y[1] positive, y[2] positive

Mu[4, 1] <- mu[5]

Mu[4, 2] <- mu[6]

Sigma[4, 1, 1] <- sigma[5]^2

Sigma[4, 1, 2] <- sigma[5]*sigma[6]*rho[4]

Sigma[4, 2, 1] <- sigma[6]*sigma[5]*rho[4]

Sigma[4, 2, 2] <- sigma[6]^2

# Priors

mu[1] ~ dnorm(0, 0.0001)

mu[2] <- mu[1]+d.mu[1]

mu[5] <- mu[1]+d.mu[3]

mu[3] ~ dnorm(0, 0.0001)

mu[4] <- mu[3]+d.mu[2]

mu[6] <- mu[3]+d.mu[4]

d.mu[1] ~ dnorm(0, 0.0001)I(0, )

d.mu[2] ~ dnorm(0, 0.0001)I(0, )

d.mu[3] ~ dnorm(0, 0.0001)I(0, )

d.mu[4] ~ dnorm(0, 0.0001)I(0, )

for (k in 1:6) {

sigma[k] ~ dunif(0, 100)

}

for(k in 1:4) {

rho[k] ~ dunif(-1, 1)

}

pi[1, 1] <- 1

pi[1, 2] <- 0

pi[1, 3] <- 0

pi[1, 4] <- 0

pi[2, 1:4] ~ ddirch(alpha[2, 1:4])

pi[3, 1:4] ~ ddirch(alpha[3, 1:4])

pi[4, 1:4] ~ ddirch(alpha[4, 1:4])

pi[5, 1:4] ~ ddirch(alpha[5, 1:4])

}"

# Data list

data.list <- with(sim.data, list(

n = nrow(sim.data),

y = cbind(log.hpv16, log.hpv18, Age_group),

alpha = matrix(nrow = 5, ncol = 4, data = 1)))

# Initial value function

inits.fun <- function() with(data.list, list(

mu = c(-1, NA, -1, NA, NA, NA),

d.mu = rep(4, 4),

sigma = rep(1, 6),

rho = rep(0, 4),

# pi = matrix(c(c(1,0,0,0),rep(0.25, 4),rep(0.25, 4),rep(0.25, 4),rep(0.25, 4)),nrow = 5, byrow = T),

.RNG.name = "base::Wichmann-Hill", .RNG.seed = sample(1:10000, 1)))

# Test model

writeLines(model.string, "model.txt")

jags.model(file = "model.txt", data = data.list, inits = inits.fun, n.chains = 1, n.adapt = 100)

# Export data to cluster

clusterExport(cl, varlist = "data.list")

# Run JAGS parallel

param.mcmc <- as.mcmc.list(parSapply(cl, X = 1:4, FUN = runjags.par,

workdir = getwd(), data = data.list, inits = inits.fun,

n.adapt = 500, n.burnin = 500, n.iter = 12500, thin = 10,

variable.names = c("mu", "sigma", "rho", "pi")))
